# Some Thoughts on the Teaching of Histology and Anatomy in Dental Colleges

**Published:** 1895-02

**Authors:** C. M. Wright

**Affiliations:** Cincinnati, O.


### Some Thoughts on the Teaching of Histology and Anatomy in Dental Colleges.

BY C. M. WRIGHT, D.D.S. CINCINNATI, O.

Read before the Ohio State Dental Society, December, 1894.

"Some thoughts" on a subject, does not imply that the writer is offering *opinions.* Thoughts may be but the shimmering vaporings of scintillating nerve cells reflexly stimulated by a commingling of present and past impressions. The present, or near sensation may arise from a bit of Roquefort at a late dinner—a *cafe noir,*—or, a lack of the usual slumber inviting night cap—and this, mixed up with stale impressions on some old memory cells, may incite imaginations or fancies, and we call these *thoughts.* Of this character are "Some thoughts on the Teaching of Histology and Anatomy in Dental Colleges."

Let us then *fancy* that knowledge can be divided into two classes: 1st. A knowledge of *facts*. 2d. A knowledge of theories (or philosophical explanations of facts—or phenomena). Patient investigators have often been fact hunters, and have contributed largely to the first division of knowledge. Logically educated scientists have explained philosophically these facts, gathered by the other workers, without ever having themselves observed a single phenomenon, and have contributed largely to our second division of knowledge. Facts sufficient to establish a theory; or facts and theories are necessary to complete knowledge.

The study of anatomy in the dissecting room, and of histology in a laboratory, has for its object two things. One is to let the student *see* the structure and structures of the human body macroscopically and microscopically. The other is to train the student in the manual art of cutting up bodies and tissues, so that they *may be seen to the best advantage*. The general surgeon also, begins the manual training of his art at the dissecting table. The dentist on the other hand begins his special manipulative training in the mechanical and operative *infirmaries* of the college.

Just here, the fancy becomes vivid, and we exclaim—What is the use to the dental student, of his course in practical dissections with the scalpel or with the microtome?

Can he not acquire morphology—all the facts that he needs, from *seeing* the dissections made, by a skilful and competent prosector or teacher. Then the qualities of textures or tissue, the minute structures are certainly much more distinctly visible in finely prepared and well mounted specimens made by experts in this art, than in the crude cuttings of the students, in a two months course.

The writer spent some time in watching the methods of a queer old teacher of medicine, some thirty years ago. His class in anatomy, consisting of twelve or thirteen disciples—men and women, sat around the "subject," with text books and diagrams in their hands, following intently the old professor, who skilfully dissected the body; explaining, lecturing and answering pertinent questions. He called the attention of the class to facts displayed by his knives and forceps, and at the same time gave philosophical explanations of the same. It was intensely interesting to the class. Three or four "subjects" dissected in this way before the class was all the "*practical anatomy*" taught in his college. The writer having studied dentistry, wondered if these students did not get more general and particular knowledge from this more Socratic method, than he himself had, by his careful dissections of the leg of a man, the arm and chest of a woman, and the foot of a negro baby. The writer as a practitioner of dentistry has been trying for thirty years to *apply* this special knowledge gained in the dissecting room to his special art-dentistry. One of the colleges in the Association of Dental Faculties is said to have no "*dissecting*," and yet teaches anatomy, and all the other colleges which have dissecting rooms and can get subjects, shrug their shoulders at the inferior (?) method of teaching anatomy. Just now the histological laboratory is the fashion in medical and dental schools, and students gather about fragments of already stained, and softened, or hardened, and imbedded tissues, and cut them into thin slices, or if they have been already cut, simply mount them on their own slides and cover them. They learn to handle bits of tissue with needles, and to put cover glasses over Canada balsam, and to paint around the edges with some protecting varnish. This is skilfully done by some students in a five or six weeks course. Just here again Fancy asks, "Would it not be as well for these students to be set down to microscopes with already mounted specimens, and with their text books, and the descriptions and explanations of the teacher, learn to *see* all that can be seen of these tissues, with various powers, low and high—these tissues prepared by those who have made a specialty of this difficult art? The time so spent by dental students would be in a line with their future work. It would be a training of the eye, a training in the art of illuminating and seeing minute objects. The technique of the laboratory for the preparation of tissues for observation can only be acquired as any other art is acquired, by tedious practice, and a few weeks spent in tinkering at a high art will not advance a student in that art. Must a man get a chisel and a mallet and chip at a block of marble for a few weeks in order that he may cultivate his taste and knowledge of sculpture? Must a man grind up paint with a pestle and mortar to become a connisseur of paintings? And, must a student cut up sections of bone, cartilage, muscle and nerve, for two months, to be an histologist? Is not the way suggested, the better one? The writer does not wish to imitate Bob Ingersoll—as an iconoclast—without offering another idol to take the place of the broken ones.

NOTE.—If this paper is deemed worthy of discussion by the members of the Ohio State Dental Society, I hope sincerely that I shall not be suspected of depreciating in the least degree the importance to us of these studies in morphology. I deem them of the utmost *use* in medical science, and the art of the dentist is but a one-sided affair, if not built up on the solid foundation of *medical science.* Physiology and pathology are as important to the dentist as to the general practitioner or any special practitioner of medicine or surgery. And these sciences are so intimately allied to morphology (or anatomy and histology) that a separation would be impossible.

